# Superparamagnetic, High Magnetic α-Fe & α″-Fe_16_N_2_ Mixture Prepared from Inverse Suspension-Polymerized Fe_3_O_4_@polyaniline Composite

**DOI:** 10.3390/polym13142380

**Published:** 2021-07-20

**Authors:** Yen-Zen Wang, Yu-Wei Cheng, Lin-Chia Ho, Wen-Yao Huang, Ko-Shan Ho, Yu-Ting Syu

**Affiliations:** 1 Department of Chemical and Materials Engineering, National Yu-Lin University of Science & Technology, Yun-Lin 640301, Taiwan; wangzen@yuntech.edu.tw; 2 Department of Chemical Engineering, Ming Chi University of Technology, New Taipei City 24301, Taiwan; louischengblue@gmail.com; 3 National Defense Medical Center, 161 Sec. 6 Minquan E. Rd., Neihu District, Taipei City 11490, Taiwan; yms40426@gmail.com; 4 Department of Photonics, National Sun Yat-sen University, 70 Lienhai Rd., Kaohsiung 80424, Taiwan; 5 Department of Chemical and Materials Engineering, National Kaohsiung University of Science & Technology, 415 Chien-Kuo Rd., Kaohsiung 80782, Taiwan; 1104311103@nkust.edu.tw

**Keywords:** polyaniline, ferrite, α″-Fe_16_N_2_, superparamagnetic, inverse suspension polymerization

## Abstract

Oleic acid (OA)-modified Fe_3_O_4_ nanoparticles were successfully covered with polyanilines (PANIs) via inverse suspension polymerization in accordance with SEM and TEM micrographs. The obtained nanoparticles were able to develop into a ferrite (α-Fe) and α″-Fe_16_N_2_ mixture with a superparamagnetic property and high saturated magnetization (SM) of 245 emu g^−1^ at 950 °C calcination under the protection of carbonization materials (calcined PANI) and other iron-compounds (α″-Fe_16_N_2_). The SM of the calcined iron-composites slightly decreases to 232 emu g^−1^ after staying in the open air for 3 months. The calcined mixture composite can be ground into homogeneous powders without the segregation of the iron and carbon phases in the mortar without significantly losing magnetic activities.

## 1. Introduction 

Recently, with the advance of technology, the requirement for fast and wireless charging becomes more and more urgent. In particular, 3C mobile electronic products need to charge within several hours using portable power sources that are usually heavy and take one or two to recharge. Therefore, we also need a fast wireless power source to solve the problem. The effective charging distance of wireless charging [1] and large-scale charging development [2], thus, becomes very important. Wireless charging can be carried out by absorbing electromagnetic (EM) waves that emit from the power generators. Additionally, we also require a stable EM wave absorbing material to transform it into power, which can be fulfilled by superparamagnetic materials with high magnetization. There are other interesting application fields related to the calcined carbon (polyaniline) coated magnetic particles [3,4,5,6] such as nanoelectronics, catalysis, optical application, biosensors, environmental remediation, energy, hydrogen storage, drug transport, magnetic resonance imaging and cancer diagnosis [7]. Metamaterial theory is also used for the applications of the magnetic materials [8].

The regular material that meets these requirements is magnetite (Fe_3_O_4_), which can be obtained from the sol-gel method [9] conducted at room temperature. Although it demonstrates a superparamagnetic property, its low magnetization (around 100 emu g^−1^) shortens the wireless charging distance. Other possible candidates with a higher magnetization than Fe_3_O_4_ are cementite (Fe_3_C) [10], ferric nitride (FexNy) [11,12] and ferrite (α-Fe) [13]; they all demonstrate saturated magnetization over 150 emu g^−1^ and even over 200 emu g^−1^ for α-Fe and α″-Fe_16_N_2_. However, α-Fe, which is the pure Fe element, is vulnerable to the O_2_ in the open air to become iron oxide.

Consequently, we need to provide some solid protection on the α-Fe, and α″-Fe_16_N_2_ itself can also provide additional protection to keep it from oxidation and maintain its high magnetization in the air.

Usually, PANIs that provide carbon and nitrogen sources can be prepared in affluent water [14,15,16,17,18] easily using water-soluble anilinium monomers. The Fe_3_O_4_ particles prepared from the sol-gel method are usually hydrophilic materials with some –OH groups on the surfaces. If it stays with anilinium monomers in the water, the prepared PANI molecules cannot cover most of the Fe_3_O_4_ particles that become very mobile in the water [19,20,21,22,23,24,25,26] due to the hydrophilicity. The obtained PANIs are not able to protect Fe_3_O_4_ particles during calcination, since most of them will stay on the surface of PANI molecules, not inside.

Inverse suspension polymerization (ISP) is usually applied to cover functional particles with polymers that can be polymerized in the water phase [27,28,29,30,31]. In this study, we are trying to prepare the protecting Fe_3_O_4_ particles by the ISP of PANIs. The sol-gel-prepared Fe_3_O_4_ particles usually own surface –OH, which can be esterificated with OA to attach some hydrophobic tails to the particles [32,33] and allow them to stably stay inside the micelles in the toluene system. Moreover, the additional aliphatic tails that come from the attaching OA can also stabilize the micelles or polymer droplets during the polymerization of anilines. In other words, hydrophilic OA-modified Fe_3_O_4_ (Fe_3_O_4_(OA)) was first mixed with the anilinium monomers in the water and became micelles in the hydrophobic toluene solvents after stirring. Eventually, the Fe_3_O_4_(OA) particles can be surrounded with long polyaniline molecules after water soluble initiators such as APS (ammonium persulfate) are introduced. The composites prepared via ISP can then be subject to calcination in the argon to transform into other iron-compounds with high magnetization.

## 2. Experimental

### 2.1. Preparation

#### 2.1.1. Synthesis of Fe_3_O_4_(OA) 

In a beaker, 7.08 g of ferric chloride hexahydrate (FeCl_3_·6H_2_O, J. T. Baker, NJ, USA) and 2.58 g of ferrous chloride tetrahydrate (FeCl_2_·4H_2_O, J. T. Baker, NJ, USA) were mixed with 40 mL of deionized water by a magnetic stirrer. The homogenized solution was transferred to a round bottom three-necked flask equipped with a water condenser in one of the mouths. One of the two remaining mouths was purged with high-purity nitrogen to prevent the oxidation of the reaction mixture at a high temperature, the other one behaved as the exhaust release outlet. The temperature of the reaction solution was ramped up to 80 °C in a silicone oil bath and kept with purging nitrogen for ten minutes. Then, 2 mL of OA (Hitachi Astemo Ltd, Tokyo, Japan) and some ammonia water (Fisher Sci., Bridgewater, NJ, USA) was introduced to tune the solution to become alkaline and then the reaction was started. The reaction continued for 30 min and was finished by stopping the stirring of the magnetic stirrer, followed by attaching a powerful magnet on the bottom of the reactor to separate the magnetic precipitate. The precipitate was washed several times with deionized water, and the clear, upper layer of the solution was discarded. The isolated black precipitate was placed in an ultrasonic oscillator for 20 min and then dried in an oven for 12 h at 60 °C, the Fe_3_O_4_(OA) was available.

#### 2.1.2. Synthesis of PANI/Fe_3_O_4_(OA) Nanocomposite 

An amount of 3 g (0.091 mol) of n-dodecylbenzenesulfonic acid (DBSA: Tokyo Kasei Kogyo Co., Tokyo, Japan) was dissolved in 50 mL of de-ionized water, the mixture was slowly stirred until a homogeneous solution was obtained, followed by the addition of 9 g (0.0968 mol) of aniline monomer (Tokyo Kasei Kogyo Co., Tokyo, Japan) and the solution was stirred to be clear. Eventually, the Fe_3_O_4_(OA) obtained from the previous experiment was added and the mixture was stirred again to become homogeneous [32,33].

A comparison emulsion polymerization prepared PANI(EB)/Fe_3_O_4_(OA) [29,30,31] was obtained in water in the absence of toluene. The resultant composite was named as PANI(EB)-Em/Fe_3_O_4_(OA).

#### 2.1.3. Calcination of PANI Nanocomposites

PANI(EB)/Fe_3_O_4_(OA) prepared in Section 2.1.2 was calcined in a tube furnace, ramping up from RT to 600–950 °C at most and staying for 30 min in the argon atmosphere. The obtained N, C-doped iron composites are named as FeNCs. When samples were kept at room temperature for two or three months in the air, they are named as FeNC-2 and FeNC-3, respectively. A sample prepared at 600 °C is named FeNC-600, etc.

### 2.2. Characterization

#### 2.2.1. Fourier Transform Infrared Spectroscopy (FTIR)

The main functional groups of neat Fe_3_O_4_(OA) and calcinated FeNC were assigned in accordance with the FTIR spectra recorded on an IFS3000 v/s FTIR spectrometer (Bruker, Ettlingen, Germany) at room temperature with a resolution of 4 cm^−1^ and 16 scanning steps.

#### 2.2.2. Ultraviolet and Visible, Near-IR Spectroscopy (UV–Vis–NIR)

The UV–Vis–NIR spectra of the PANI(ES) (PANI without dedoping by NH_4_OH_(aq)_) in the PANI(ES)/Fe_3_O_4_(OA) nanocomposites were obtained from a Hitachi U-2001 and DTS-1700 NIR Spectrometer (Nicosia, Cyprus). The wavelength ranged from 300 to 1600 nm.

#### 2.2.3. TGA (Thermogravimetric Analysis)

The mass loss percentages of neat PANI(EB) and PANI(EB)/Fe_3_O_4_(OA) upon calcination (thermal degradation) were monitored and recorded using TGA (TA SDT-2960, New Castle, DE, USA) thermograms.

#### 2.2.4. Scanning Electron Microscopy (SEM)

The sizes and morphologies of neat Fe_3_O_4_(OA), non-calcinated PANI(EB)/Fe_3_O_4_(OA), and calcinated FeNCs were characterized using SEM (field emission gun scanning electron microscope, AURIGAFE, Zeiss, Oberkochen, Germany).

#### 2.2.5. Transmission Electron Microscopy (TEM)

Samples, of which photos were taken using the field emission transmission electron microscope, HR-AEM (HITACHI FE-2000, Hitachi, Tokyo, Japan), were first dispersed in acetone and put on carbonic-coated copper grids dropwise before subjecting to the emission.

#### 2.2.6. Raman Spectroscopy

The Raman spectra of calcinated PANI(EB)s and FeNCs treated at different temperatures were obtained from a Raman spectrometer (TRIAX 320, HOBRIA, Kyoto, Japan).

#### 2.2.7. Powder X-ray Diffraction (Powder XRD)

A copper target (Cu-Kα) Rigaku X-ray source (Rigaku, Tokyo, Japan) generating X-ray with a wavelength of 1.5402 Å after electron bombarding was used to create the diffraction patterns of neat Fe_3_O_4_(OA) and FeNCs. The scanning angle (2θ) that ranged from 10° to 70° with a voltage of 40 kV and a current of 30 mA, operated at 1° min^−1^.

#### 2.2.8. X-ray Photoelectron Spectroscopy (XPS)

The binding energy spectra of Fe 2p of FeNCs treated at different temperatures were used to characterize the characteristic crystallization planes of α–Fe, Fe_3_C, FeNx and, Fe_3_O_4_ after calcination, and were obtained from an XPS instrument of Fison (VG)-Escalab 210 (Fison, Glasgow, UK) using an Al Ka X-ray source at 1486.6 eV. The pressure in the chamber maintained was under 10^−6^ Pa or lower during performance. Tablet samples were prepared by pressing in a stapler with a ring mold.

#### 2.2.9. Superconductor Quantum Interference Device (SQUID)

The paramagnetic properties of neat Fe_3_O_4_(OA) and various FeNCs were measured from a SQUID of Quantum Design MPMS-XL7 (San Diego, CA, USA)

## 3. Results and Discussion

### 3.1. FTIR Spectra

**Figure 1:**
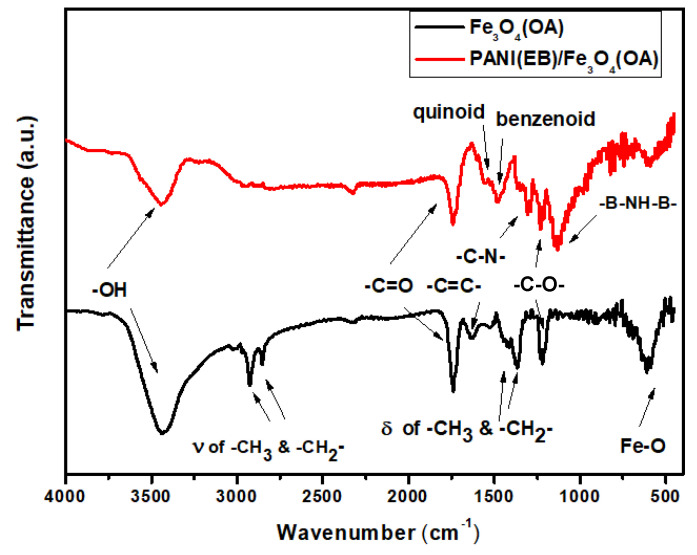
FTIR-spectra of neat Fe_3_O_4_(OA) and PANI(EB)/Fe_3_O_4_(OA) composite.

The hydrophilicity of Fe_3_O_4_(OA) nanoparticles comes from the hydroxylated surfaces created during the sol-gel process, assigned at ~3300 cm^−1^ according to Figure 1. Some of the hydroxyl groups are still present after esterification with OA, as described in Figure 1 as well, indicating that some of the –OH groups remained intact after esterification and polymerization. The remaining hydrophilicity of the nanocomposite makes it still dispersible in micelles before the polymerization mixing with anilinium monomers or staying in polymer droplets after polymerization, which was randomly dispersed in the hydrophobic toluene matrix. The polymerization of anilinium monomers after the addition of water-soluble initiator APS in the presence of Fe_3_O_4_(OA) did not destroy the carbonyl groups of the ester that link the OA onto the Fe_3_O_4_ surface of the nanoparticles either, illustrating that the long hydrocarbon tails of the OA are still firmly connected to the Fe_3_O_4_ nanoparticle’s surface. It provided the nanocomposites with some hydrophobicity and affinity to the toluene and the large polymer droplets were still able to suspend in the solvent after polymerization. The polymer droplets need to de-emulsify with acetone before collecting the nanocomposite products through filtration. The symmetric and asymmetric stretching mode of aliphatic methylene and the methyl groups of OA tails assign at 2920 and 2840 cm^−1^, respectively. The additional peak around 587 cm^−1^ reveals the presence of the Fe–O bonding of both the neat Fe_3_O_4_(OA) and the nanocomposite, revealing that Fe_3_O_4_(OA) nanoparticles are staying inside the nanocomposite, even after de-emulsification and filtration. The assignments of the main functional groups of polyaniline and Fe_3_O_4_(OA) are listed in Table 1 and illustrated in Figure 1.

The obtained PANI(ES)/Fe_3_O_4_(OA) nanocomposites were then dedoped into PANI(EB)/Fe_3_O_4_(OA) in NH_4_OH_(aq)_ and its FTIR-spectrum is also demonstrated in Figure 1. The feature functional groups of PANI(EB) can be found for PANI(EB)/Fe_3_O_4_(OA) in Table 1 and Figure 1, revealing that the polymerization did not change the characteristic functional groups of PANI in the presence of Fe_3_O_4_(OA).

### 3.2. λ_max_ of PANI in the Nanocomposite Obtained from UV–Vis–NIR Spectra

**Figure 2:**
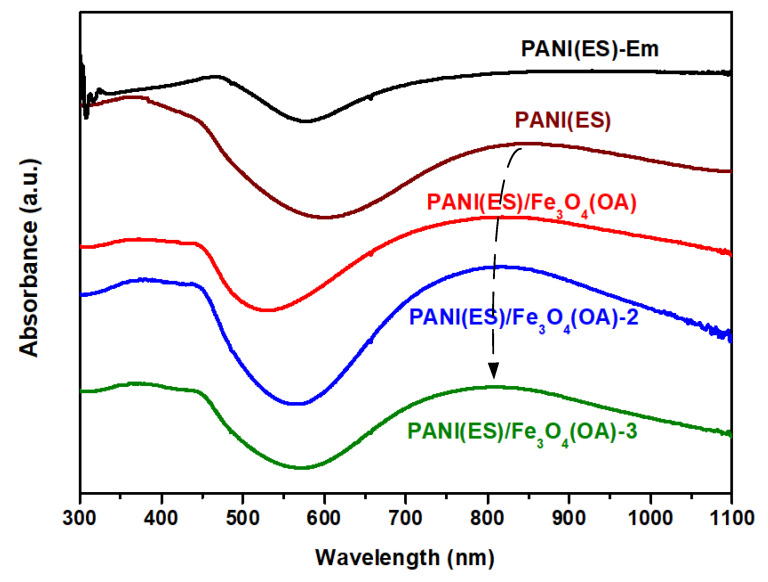
UV–Vis–NIR spectra of PANI(ES) and various PANI(ES)/Fe_3_O_4_(OA) composites.

**Scheme 1:**
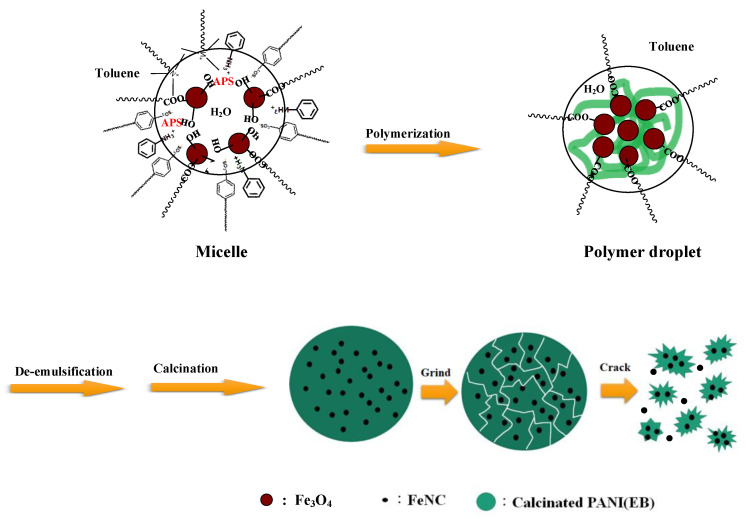
Preparing diagram of FeNCs via inverse suspension polymerization and calcination.

If most of the Fe_3_O_4_(OA) in the nanocomposite is completely covered or embedded in the PANI matrix, it can also induce the shifting of the λ_max_ of PANI in the UV–Vis spectra due to the interaction. Figure 2 illustrates the flattening effect on the λ_max_ in the NIR region, the so-called free carrier-tail [14,15,16,17,18] in this region for the PANI(ES)-Em prepared via emulsion polymerization. It also represents a nanofibrous morphology that contributes to the carrier-tail due to the highly extended conjugation chain length. However, in accordance with Figure 2, the PANI(ES) prepared via ISP does not demonstrate any free carrier-tail, but the significant λ_max_ peak and the morphology is not fibrous either, which will be confirmed in the TEM micropicture. The λ_max_ of PANI(ES) prepared by ISP is around 860 nm, which is also in the near-IR region, indicating that its conjugation chain length is still long, and the chain is still extended. After the Fe_3_O_4_(OA) nanoparticles were introduced before the beginning of the polymerization, the λ_max_ of PANI(ES) blue-shift from 860 to 800 nm with increasing Fe_3_O_4_(OA) nanoparticles and curve became more bent. The blue shift and the presence of the bended curves of the nanocomposites reflected the shortening of the conjugation of the PANI molecules whose extensions were slightly recoiled. Since the λ_max_ is still high at 810 nm, a random-coil morphology (λ_max_ = 780 nm) is not thought to be present. The attracting force that causes the recoiling of the PANI(ES) molecules is believed to stem from the formation of the H-bonding between the left –OH groups and the amino groups of PANI(ES). The blue-shifting is enhanced when more Fe_3_O_4_(OA) is introduced, referring to Figure 2. The ISP approach effectively polymerizes the anilinium monomers inside micelles where lots of Fe_3_O_4_(OA) nanoparticles are already present. It is believed that some of the H-bonds are already formed before the addition of water-soluble APS initiator and most of the Fe_3_O_4_(OA) nanoparticles inside micelles can be surrounded and protected by both monomers before polymerization and polymers after polymerization, which is described in Scheme 1. The presence of the long aliphatic/long tail of Fe_3_O_4_(OA) can improve the stability of the micelles in the toluene solvents as well and less CTAB is necessary to create the inverse micelle (W/O). Moreover, the hydrophobic tails of Fe_3_O_4_(OA) are extended to the toluene phase and can also immobilize the Fe_3_O_4_ inside water micelles, allowing the growing PANI molecules to entangle around them.

When PANI is prepared via common emulsion polymerization, most of the Fe_3_O_4_(OA) particles will randomly distribute in the water and the fibrous PANI is created inside the micelles where less water is present. Eventually, the obtained nanofibrous PANI molecules can only attract lots of Fe_3_O_4_(OA) particles on the surface through H-bonding, which will be seen in the SEM micropicture. The Fe_3_O_4_(OA)-covered fibrous PANI surely cannot form a stable/high magnetic FeNC compound after calcination, since the formed high magnetic materials cannot be protected by the PANI during calcination.

### 3.3. TGA Thermogram

**Figure 3:**
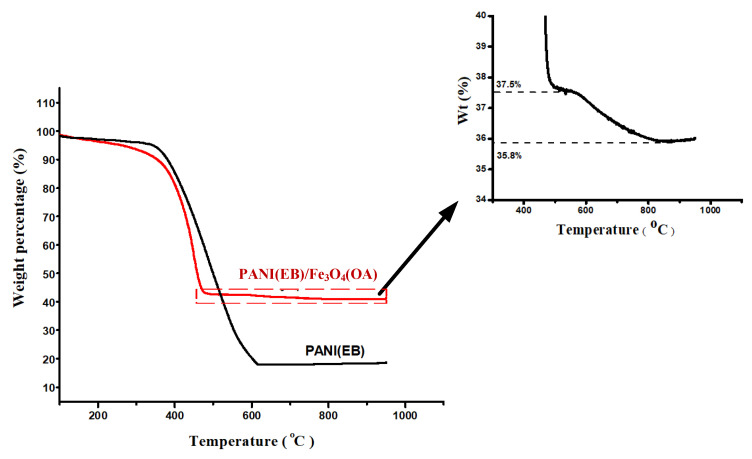
TGA thermograms of PANI(EB) and PANI(EB)/Fe_3_O_4_(OA).

The mass loss and rate of the calcination at a high temperature can be monitored by the weights vs. temperatures in the thermogram demonstrated in Figure 3. The neat PANI(EB) experienced significant weight loss after 400 °C, which originated from the crosslinking of the neighboring molecules [18], and the weight loss continued gradually until 600 °C, when the crosslinking finished, and carbonization started. Almost no mass loss occurred after 600 °C for PANI(EB), which is the reason why the lowest calcination temperature chosen was 600 °C, after which the weight loss was entirely contributed from the PANI(EB)-covered iron-compounds not from PANI(EB) only, in accordance with Figure 3. If we check the difference of the residue weights of neat PANI(EB) after 600 °C and weight of PANI(EB)/Fe_3_O_4_(OA) at 600 °C from Figure 3 and Table 2, it is 22.4 wt% (35.9% − 13.5% = 22.4%). It means there is about 22.4 wt% of iron-compound in the composite, which is thermally stable until 600 °C. There is only 1.7 wt% loss (37.5% − 35.8% = 1.7%) after 600 °C for the composite, as seen in the inset of Figure 3, which is all contributed from the thermal degradation of the FeNCs from 600 to 950 °C. The actual degrading % for FeNC is around 1.7/22.4 ≈ 7.6 wt%. The composition variation and the types of atoms (Fe, N, C, or O) that are lost during the calcination of the FeNCs from 600 to 950 °C are strongly related to the magnetic activity, which will be characterized from the powder X-ray diffraction patterns, XPS, and SQUID spectra.

### 3.4. SEM Micropicture

**Figure 4:**
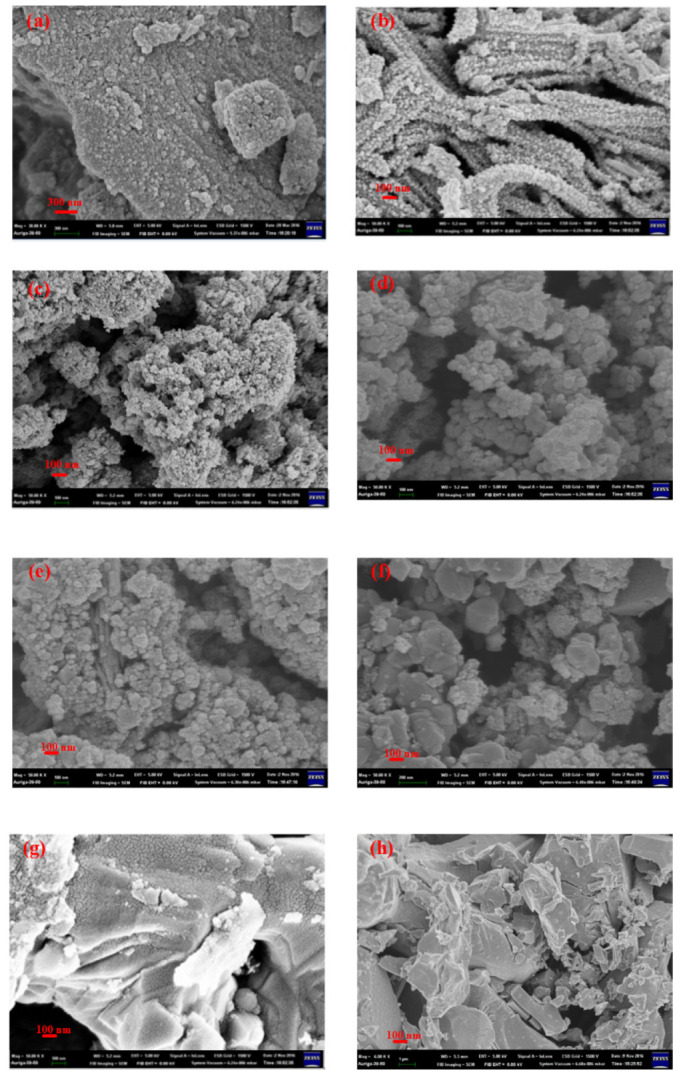
SEM micropictures of (**a**) Fe_3_O_4_(OA), (**b**) PANI(EB)-Em/Fe_3_O_4_(OA), (**c**) PANI(EB)/Fe_3_O_4_(OA), (**d**) FeNC-650, (**e**) FeNC-700, (**f**) FeNC-800, (**g**) FeNC-900, (**h**) FeNC-950.

Unlike the neat Fe_3_O_4_ particles that demonstrate a pearl-like morphology, the Fe_3_O_4_(OA) particles are found to be able to coagulate by the interactive long, aliphatic chains of OA and become the huge cake-like morphology in Figure 4a after being treating with OA via esterification. The PANI(EB) prepared in the presence of Fe_3_O_4_(OA) particles via common emulsion polymerization demonstrates rib-like, juxtaposed nanofibers fully covered with lots of Fe_3_O_4_(OA) particles on their surfaces in Figure 4b, which is commonly seen in the traditional emulsion polymerization of PANI [31,34]. These exposed Fe_3_O_4_(OA) particles on the fibrous PANI(EB) certainly can transform or convert to other iron-compounds with higher magnetic activity. However, the formed iron-compounds would be directly exposed to the O_2_ in the atmosphere at RT without any protection. Therefore, these iron-compounds would easily oxidize and recover to iron-oxide, whose magnetic force is far below that of the FeCx, FeNx, or α-Fe obtained after calcination. Consequently, an ISP system is designed to encapsulate the un-protected Fe_3_O_4_(OA) particles with PANI(EB) that can develop into a dense, strong protecting carbon layer after calcination at high temperature through crosslinking and carbonization.

The –OH groups of the sol-gel-prepared Fe_3_O_4_(OA) stay inside the micelles before polymerization. Additionally, after the polymerization in the inverse suspended system, most of the PANIs are formed in the micelles and become polymer droplets that then coalesce and develop the morphology seen in Figure 4c when the ISP system was eventually de-emulsified by the addition of acetone (the breaking of polymer droplets) and the fibrous morphology is never seen.

Upon calcination at high temperatures, the Fe_3_O_4_(OA) that covered with PANI(EB) would convert to other magnetic materials with a higher magnetic activity. Combining the increasing magnetic attractive forces and thermal energy provided by the high temperature calcination, these Fe_3_O_4_(OA) nanoparticles were able to transform and to merge into bigger particles due to the increasing magnetic attraction forces with increasing temperatures, as seen from Figure 4d–h. The sizes of the new particles are smaller when the calcination temperatures are below 700 °C. Bigger cake-like ensembles are perceivable when temperatures reach 800 °C. These cake-like ensembles even impinge further into huge slabs around 900 °C, revealing the occurrence of extremely high magnetic attractive forces after 900 °C. The types of magnetic materials created when Fe_3_O_4_(OA) are calcined inside of the PANI(EB) matrix at high temperatures can be studied by checking their X-ray diffraction patterns and XPS spectra.

### 3.5. TEM Micropicture

**Figure 5:**
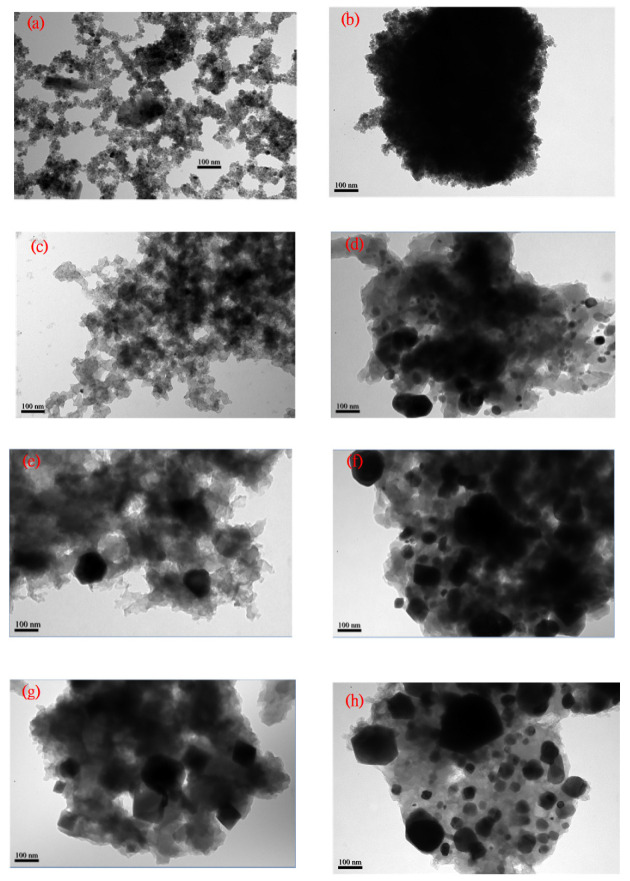
TEM micropictures of (**a**) Fe_3_O_4_(OA), (**b**) PANI(EB)/Fe_3_O_4_(OA), (**c**) FeNC-600, (**d**) FeNC-650, (**e**) FeNC-700, (**f**) FeNC-800, (**g**) FeNC-900, (**h**) FeNC-950.

The pearl-like chain morphology of the TEM micropicture in Figure 5a expresses the neat Fe_3_O_4_(OA) particles that are connected to each other by the inter-entangled or inter-digitized long alkyl tails of the attached OA. Most of the Fe_3_O_4_(OA) particles were covered by the PANI(EB) after the ISP at RT, as seen in Figure 5b, where only some tiny ones can be seen in the margins of the big ensemble. The pretty dark and homogeneous color seen in Figure 5b reveals that Fe_3_O_4_(OA) particles are uniformly distributed in the PANI(EB) matrix. When the temperature reached 600 °C, most of the PANI(EB) were thermally degraded and only 13.5 wt% were left, according to Figure 3 and Table 2, the sample became more transparent in Figure 5c, and some tiny Fe_3_O_4_(OA) nanoparticles transformed and coalesced into bigger, dark particles after 600 °C. Whether they still remain in the form of Fe_3_O_4_ and what kind of new iron-compounds formed after 600 °C, can be analyzed using X-ray or XPS spectra. The particle-assembly phenomena were enhanced with the calcination temperature and huge grain-like particles developed from 600 to 950 °C, in accordance with Figure 5c–h. The growing size of the dark particles resulted from the increasing magnetic attractive forces with temperature, which originated from the formation of some iron-compounds with a high magnetic activity. There are N, C, and O atoms inside the nanocomposite, except Fe. Additionally, the FeNCs are all derivatives of Fe_3_O_4_(OA) via calcination. The increasing magnetic forces compared to the neat PANI(EB)/Fe_3_O_4_(OA) or the neat Fe_3_O_4_(OA) can be attributed to the newly formed FeCx, or α-Fe, even the α″-Fe_16_N_2_ compounds that own much higher magnetic forces than the neat Fe_3_O_4_(OA). Furthermore, the obtained Fe_3_C (cementite) is the most stable FeCx compound. The variation % of these atoms and types of iron-compounds in the composite with increasing temperatures can be understood from either the XRD patterns or the XPS for each compound.

**Figure 6:**
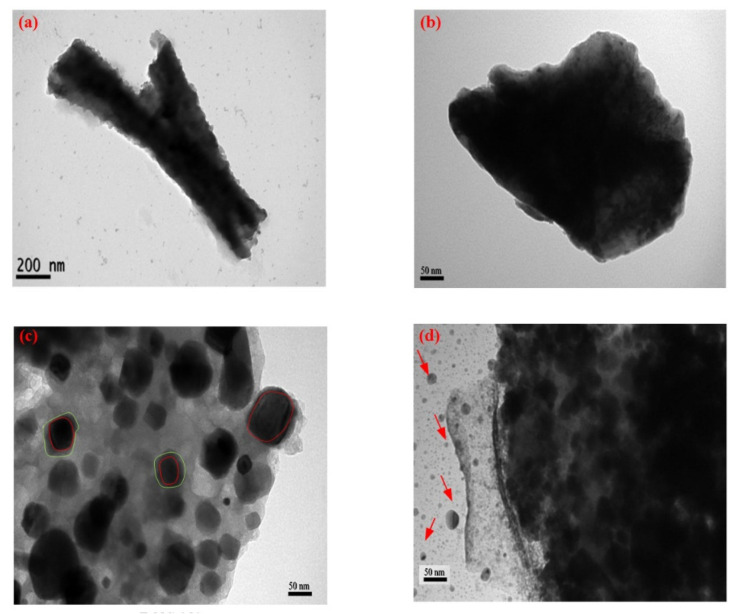
TEM micropictures of (**a**) PANI(EB)-Em/Fe_3_O_4_(OA), (**b**) PANI(EB)/Fe_3_O_4_(OA), (**c**) FeNC-950, (**d**) Ground FeNC-950.

The juxtaposed fibrous PANI(EB), fully covered with Fe_3_O_4_(OA) nanoparticles (PANI(EB)-Em/Fe_3_O_4_(OA)), can also be seen in Figure 6a. These fibers, which merged into a huge ensemble, gradually became hard stone in Figure 6b and cannot be easily broken into small pieces by simply grinding them in the mortar. It is believed that the surface Fe_3_O_4_(OA) nanoparticles developed into hard covering materials and the calcined PANI(EB) (950 °C) mostly remain inside the composites. In contrast, the ISP-prepared composite allowed the inclusive Fe_3_O_4_(OA) nanoparticles to develop into individual small magnetic particles inside the FeNCs, as seen in Figure 6c, which can be easily ground into tiny particles in the mortar, as depicted in Scheme 1. Amazingly, some highly crystallized stone powders can be released from the cracked particles after grinding, as seen in Figure 6d, which is also illustrated in Scheme 1. Actually, the breaching by grinding occurs following the carbonized PANI(EB) boundaries. It means we are able to fabricate any shapes of high magnetic stones by further sintering these magnetic powders in different shapes of molds at a temperature far below the melting points of the magnetic stones. Moreover, the magnetic powders can also be protected from oxidation during sintering by the carbonized coverings.

### 3.6. Raman Spectra

**Figure 7:**
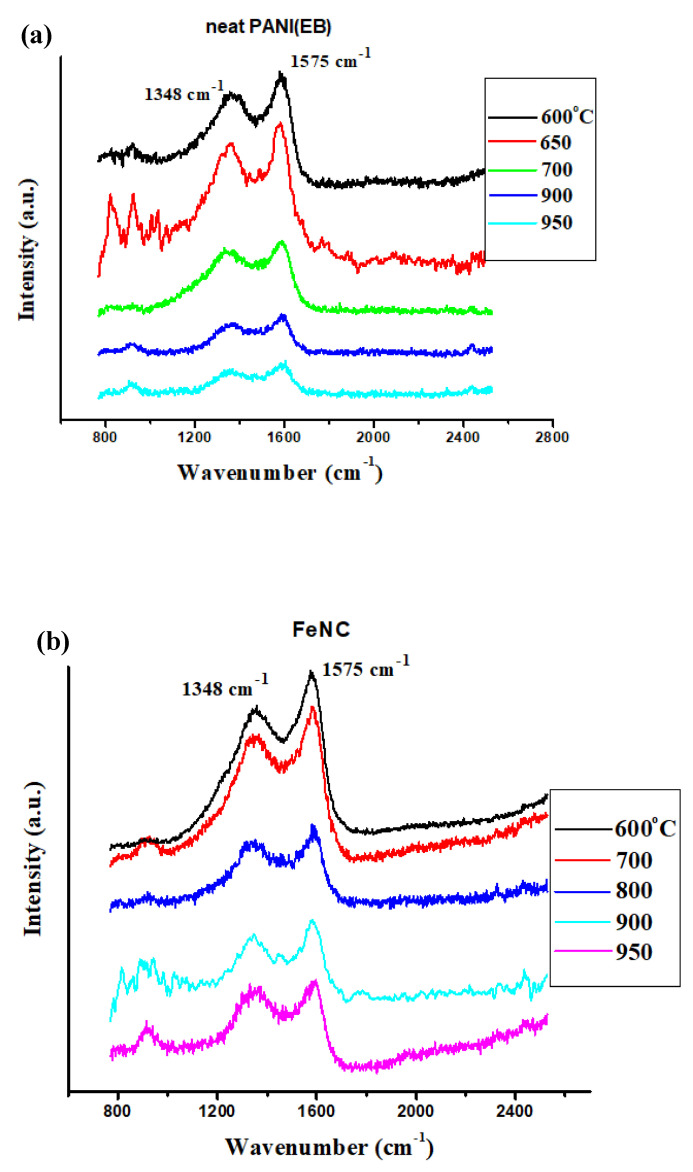
Raman-spectra of (**a**) neat PANI(EB) (**b**) FeNCs.

The ratio (I_D_/I_G_) of –C–C– single bonds (sp^3^, D-band) with –C=C– double bonds (sp^2^, G-band) demonstrated in the Raman spectrum can be used to monitor the degree of the thermal degradation of organic compounds. When it increases with temperature (more G-bands thermally destroyed to become D-band carbons), it means a rougher surface structure is created after heating and vice versa. The I_D_ and I_G_ peaks assigned at 1348 and 1575 cm^−1^ in the Raman spectrum represent the D- and G-band of the covalent-bonded carbons, respectively. The surface structures that vary with the calcination temperatures for PANI(EB) and FeNCs are monitored using the Raman spectra in Figure 7 and their I_D_/I_G_ values are listed in the last two columns of Table 2. The I_D_/I_G_ of PANI(EB) in Figure 7a decrease with the calcination temperature due to the crosslinking and some degree of the ordering of the graphene lattice, referring to the PANI(EB) matrix structure of the FeNC composites that is actually becoming more and more smooth with the formation of plane sp^2^ (C=C) bonding. However, the entire composite does not follow that trend with increasing temperatures when Fe_3_O_4_(OA) is present. The degradation of OA tails and the thermal transformation of the iron-compounds, which accompany the formation of bonds between Fe and N, or the C atoms of the PANI(EB), can destroy more C=C bonding too since it owns more active π-bonds. Moreover, the iron-compound itself would experience a crystallographic transformation at high temperatures in accordance with the Fe–C phase diagram after 900 °C. All these possible newly formed bonds and transformations at high temperatures play significant roles in the eventual structures of the FeNCs obtained at different temperature calcination, resulting in the increasing I_D_/I_G_ with temperature in Figure 7b and Table 2. Certainly, the iron-compounds obtained at various temperatures all demonstrate much higher magnetic forces than the starting material PANI(EB)/Fe_3_O_4_(OA), which will be illustrated later.

### 3.7. Powder XRD Patterns

**Figure 8:**
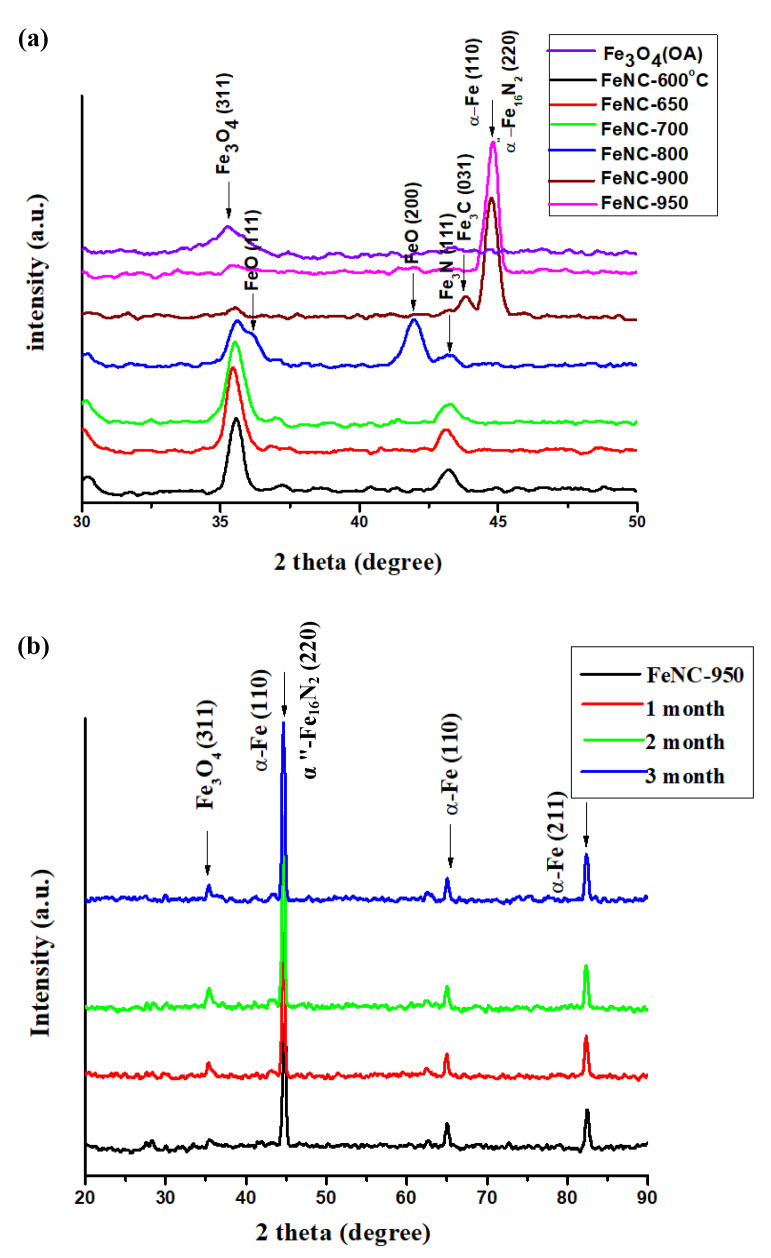
X-ray diffraction patterns of (**a**) FeNCs and (**b**) FeNC-950 staying in the open air for months.

The powder XRD patterns of neat Fe_3_O_4_ and FeNC treated at the various temperatures displayed in Figure 8a significantly demonstrates the variation of the characteristic crystalline plane-peaks from Fe_3_O_4_ to FeO, Fe_3_C, Fe_3_N, and α-Fe (α″-Fe_16_N_2_) with temperature. In particular, when the temperature was close to 900 °C, α-Fe and α″-Fe_16_N_2_ became the dominant core product due to the migration of O, N, and C atoms out of the core materials. Other type of hard, stable iron-compounds such as Fe_3_C or Fe_3_N with lower magnetic activities formed the covering materials protecting the inner α-Fe from oxidation and losing magnetic activity in the atmosphere at RT. The presence of α″-Fe_16_N_2_ in the core also provided additional protection for the formed α-Fe. The characteristic diffraction patterns of α-Fe(110) and α″-Fe_16_N_2_(220) illustrated in Figure 8b remain almost intact after staying in the air for 3 months, which will otherwise become iron oxides in less than 1 month without any protection for neat α-Fe (ferrite). It again illustrates the necessity and importance of covering iron-materials with PANI(EB) via inverse suspension polymerization.

According to FWHM, the crystallite sizes for FeNC-900 and -950 are 1.15 and 3.75 nm, respectively (calculated from the X-ray diffraction patterns of Figure 8b α-Fe(110) peak), which are much smaller than TEM (Figure 6c) The size of α-Fe (or α″-Fe_16_N_2_) crystallites also increased with temperature from 900 to 950 °C. The size of Fe_3_C based on Fe_3_C (031) plane in Figure 6c is 3.65 nm, which is also much smaller than the particles illustrated in TEM (Figure 6c).

### 3.8. XPS Spectra

**Figure 9:**
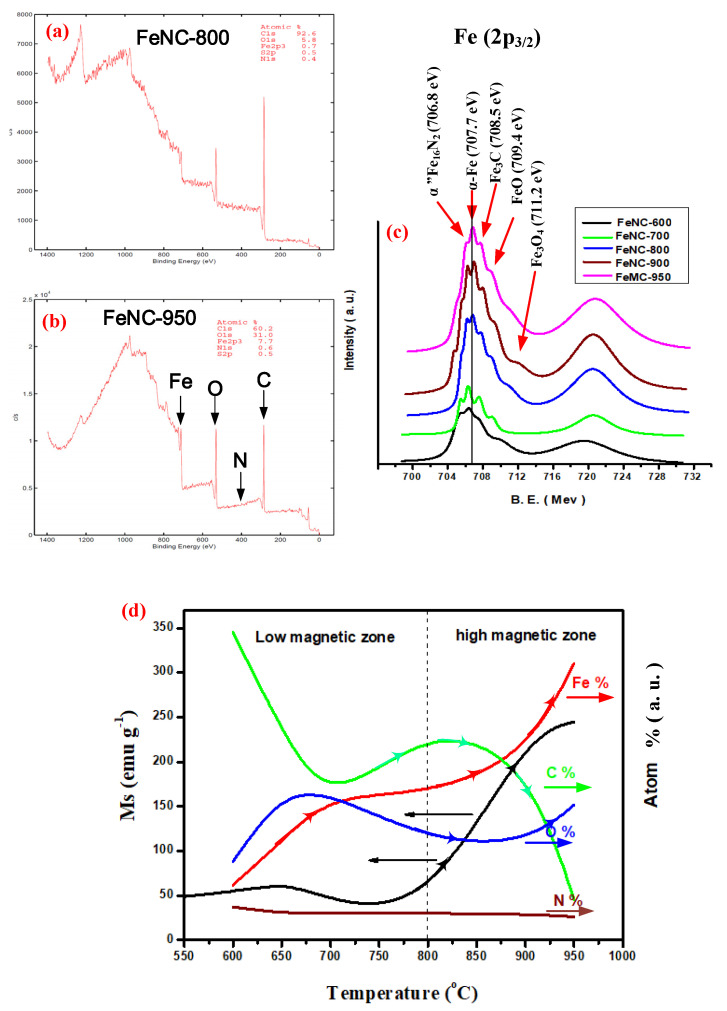
XPS spectra of (**a**) FeNC-800, (**b**) FeNC-950, (**c**) Fe (2p_3/2_) of FeNCs, (**d**) specific magnetization (Ms) and various atom % of FeNCs at different temperatures.

**Figure 10:**
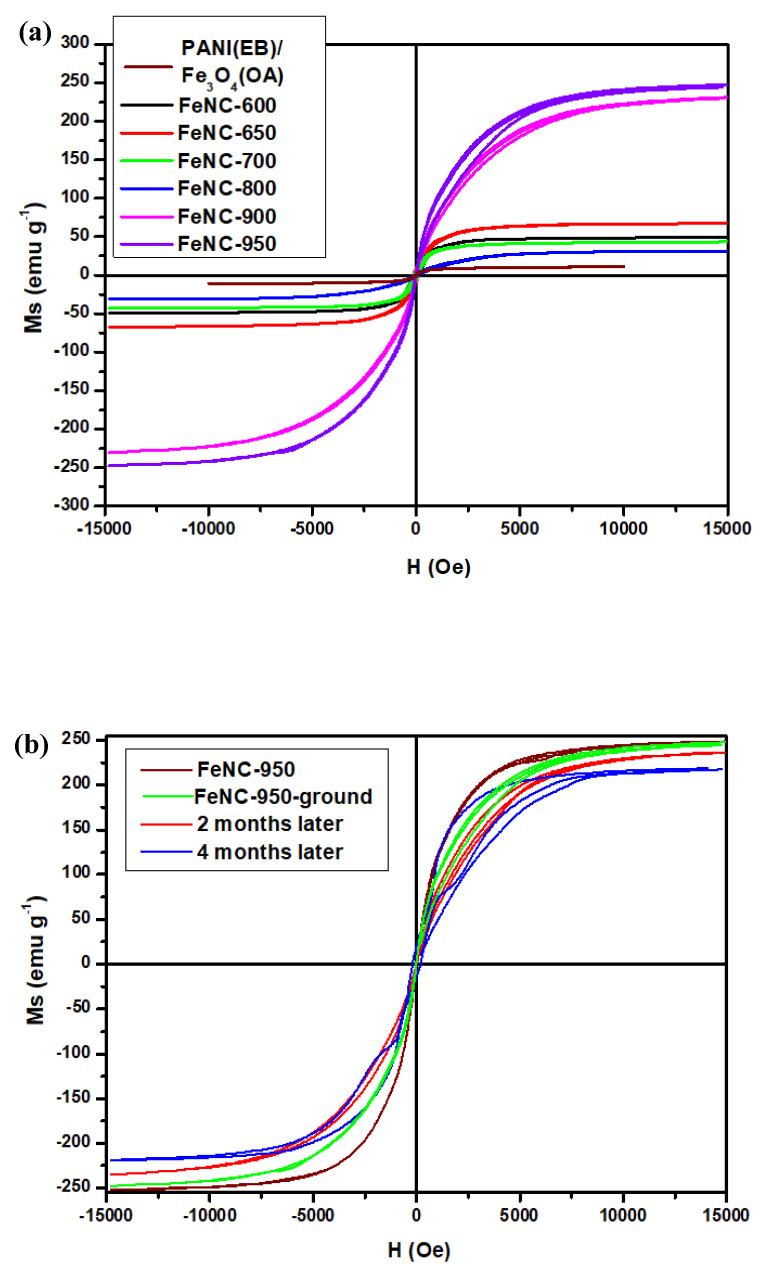
Saturated magnetization of (**a**) FeNCs and (**b**) various FeNC-950.

Typical XPS spectra of FeNCs recording % of Fe, N, C, and O atoms in Figure 9a,b provide the % variation of each atom at different temperatures. Additionally, the Fe(2p_3/2_) of the composites calcined at various temperatures was revealed in Figure 9c. The characteristic peaks of α″-Fe_16_N_2_ (706.8 eV), α-Fe (707.7 eV), Fe_3_C (708.5 eV), Fe_3_O_4_ (710.8 eV), and FeO (709.4 eV) [35,36,37,38,39], respectively, were illustrated in Figure 9c as well. Referring to the magnetic data obtained from Figure 10, we are able to construct Figure 9d, which illustrates how magnetic activity and each type of atom % varied with the calcination temperatures. The positions of the Fe(2p_3/2_) patterns, which ranged from 700 to 714 MeV, can be applied to understand what kind of iron-compounds the ISP-prepared PANI(EB)/Fe_3_O_4_(OA) composite became during calcination. Additionally, the satellite peaks that shift from ~715 up to ~725 eV when the thermal treatment temperature increases from 600 to 950 °C correspond to the ferrous compounds [40,41]. According to Figure 9c, α-Fe was not the dominant compound until the calcination temperature raised over 800 °C, but Fe_3_C is already created above 700 °C by a reaction between the included Fe_3_O_4_(OA) nanoparticles and the surrounding PANI(EB) matrix seen in Figure 9d and exists for all calcination temperatures. In other words, Fe_3_C, which is a very stable, hard magneton, dominates in the composite in the beginning of calcination and α-Fe formed later by driving some C atoms out of the core area at a temperature higher than 800 °C. Therefore, we can find a clear and sharp increase in the Fe % and a deep decease in the C % when the temperature was over 800 °C in Figure 9d, which was also accompanied with a sudden increase in magnetic forces. The formation of an α-Fe core and an Fe_3_C shell provides a facile way to fabricate stable magnetons with ultrahigh SM values, which will be discussed in the following section.

### 3.9. SQUID Spectra

Ferrite (α-Fe) and α″-Fe_16_N_2_ are two of the magnetic materials with SM over 200 emu g^−1^, α″-Fe_16_N_2_ even reaches 300 emu g^−1^. Limited to its vulnerable structure, α-Fe easily fuses with O, C, or N atoms to become iron oxide, Fe_3_C, and FeNx, respectively. Figure 10a clearly indicates that the SM of ISP-prepared PANI(EB)/Fe_3_O_4_(OA) varies with calcination temperatures from 600 to 950 °C. The SM increases from 10 to 249 emu g^−1^ when calcination increases from RT to 950 °C, in accordance with Figure 10a, due to the transformation from Fe_3_O_4_ to α-Fe and α″-Fe_16_N_2_, which has already been proven using the X-ray and XPS spectra discussed in the previous sections. However, the SM does not monotonously increase with temperature since different iron-compounds with a higher or lower SM formed at different calcination temperatures. The SM slightly increased from 10 to 67 emu g^−1^ at 650 °C and fell back to 27 emu g^−1^ after the temperature increased to 800 °C. Briefly, the SM does not exceed 70 emu g^−1^ if calcination maintains below 800 °C. The X-ray patterns in Figure 8a and the XPS spectra in Figure 9c demonstrated the presence of mixtures of Fe_3_O_4_, FeO, and Fe_3_C and their SMs are well below 250 emu g^−1^ theoretically. Until 900 °C is reached, the SM abruptly raises to 230 emu g^−1^ and then 245 emu g^−1^ at 950 °C. Again, their X-ray patterns and XPS spectra illustrate the formation of affluent α-Fe and α″-Fe_16_N_2_ at this stage by driving other atoms out of the core area with the help of high thermal energy. Certainly, there might be the presence of the lattice transformation of the iron-compounds after 900 °C, which also propels N, C, and O atoms to the outer area of α-Fe and α″-Fe_16_N_2_ to become protecting shell materials. Moreover, α″-Fe_16_N_2_ is able to prevent α-Fe from oxidation as well. According to the common Fe–C diagram, BCC-Fe would convert to FCC-Fe after 912 °C. Therefore, it is very possible for the iron compounds to undergo a significant atom rearrangement when the temperature is over 900 °C.

Under the protection of these stable iron-compounds, the SM of a mixture of α-Fe and α″-Fe_16_N_2_ decays only to 232 emu g^−1^ after staying in the air for 3 months, referring to Figure 10b. The slight decrease in the SM of FeNC-950 can be attributed to the possible surface oxidation of the powders during grinding, which largely created the surface area. All the FeNCs demonstrate superparamagnetic behaviors, referring to Figure 10a.

The ground FeNC-950 sample in the mortar retains the high SM of 245 emu g^−1^, as shown in Figure 10a. The FeNC prepared from the calcination of ISP-prepared PANI(EB)/Fe_3_O_4_(OA) is unlike other magnetic materials with a high SM, it can be easily broken into small pieces by a low degree of milling without losing the SM. The breaching points or parts of FeNC by grinding or milling are believed to follow the carbonized PANI(EB) boundaries that are affluent with carbon material, as illustrated in Scheme 1. In other words, FeNC is composed of small particles made of α-Fe and α″-Fe_16_N_2_ covered with carbon materials, which is very similar to the TEM micropicture in Figure 6c.

## 4. Conclusions

Fe_3_O_4_ nanoparticles were successfully and fully covered by polyanilines via inverse suspension polymerization in accordance with the SEM and TEM micrographs and the nanoparticles were able to develop into magnetons protected by the carbonization materials developed from polyaniline at different calcination temperatures. The saturated magnetization of the calcined iron-composites slightly increased from RT to 700 °C first and depressed continuously until 800 °C. A surprising jump of the saturated magnetization was found during 800~900 °C calcination. Based on the spectra of the X-ray diffraction and the XPS of iron-compounds calcined at temperatures higher than 900 °C, we understand a mixture of α-Fe and α″-Fe_16_N_2_ was formed in the core area and protected by the surrounding hard iron-compounds such as cementite (Fe_3_C). The composite obtained from calcination at 950 °C slightly lost its saturated magnetization from 245 to 232 emu g^−1^ after staying for 3 months in the air. Moreover, unlike the common high magnetic materials, the calcined high magnetic product is not too hard to break by grinding or milling and does not cause the loss of saturated magnetization either. The easily broken properties do not originate from the presence of the cementite, since its hardness is also very high, but from the weak surrounding carbonized materials developed from polyaniline. For the time being, the application of inverse suspension polymerization to cover iron-magnetic materials with polymers before subjecting them to calcination to prepare the high magnetic materials proves to be very successful.

In the future, sintering approaches will be applied to shape the ground magnetic powders into various shapes of magnetons with high saturated magnetization in specific molds for different purposes.

## Figures and Tables

**Table 1:** Assignments of FTIR spectra.

–OH	3400 cm^−1^
–CH_3_, –CH_2_ (stretching)	2900, 3000 cm^−1^
–C=O	1750 cm^−1^
–C=C–	1650 cm^−1^
Quinoid ring	1570 cm^−1^
Benzoid ring	1476, cm^−1^
–CH_3_, –CH_2_ (bending)	1400, 1350 cm^−1^
–C–N–	1307 cm^−1^
–C–OH	1250 cm^−1^
–B–NH–B–	1135 cm^−1^
Fe–O	590 cm^−1^

**Table 2:** Various properties of FeNCs vs. temperature.

	Properties	Ms ^(a)^ (emu g^−1^)	Weight ^(b)^ (%)		I_D_/I_G_ ^(c)^	
T (°C)			PANI(EB)	FeNC	PANI(EB)	FeNC
Room Temp.		17.7	100	100 (PANI(EB)/Fe_3_O_4_(OA))	1.32	1.30 (PANI(EB)/Fe_3_O_4_(OA))
600		49.3	15.3	37.2	0.96	0.89
700		43.2	13.0	36.5	0.86	0.95
800		31.0	13.1	36.0	0.82	0.97
900		229.1	13.3	35.9	0.79	0.98
950		244.9	13.5	35.9	0.74	0.98

^(a)^ Saturation magnetization at various temperatures obtained from SQUID; ^(b)^ Weight percentages at various temperatures obtained from TGA thermograms; ^(c)^ Intensity of D-band over G-band at various temperatures, obtained from Raman spectra.
